# The Reaction Mechanism Study for the F_3_ System

**DOI:** 10.1155/2022/7088063

**Published:** 2022-04-28

**Authors:** Dequan Wang, Nan Gao, Hongmei Yu, Yuxuan Bai, Jing Cao, Chunmei Hu, Yanchun Li, Huiling Liu, Xuri Huang

**Affiliations:** ^1^Laboratory of Theoretical and Computational Chemistry, Institute of Theoretical Chemistry, Collage of Chemistry, Jilin University, Changchun, China; ^2^Department of Thoracic Surgery, China-Japan Union Hospital of Jilin University, Changchun, China; ^3^High Performance Computing Center of Jilin University, China; ^4^College of Life Science, Sichuan Agricultural University, Ya'an, China; ^5^Hematology and Oncology Department, The Second Hospital, Jilin University, Changchun, China

## Abstract

In order to study the F_3_ system, an accurate global adiabatic potential energy surface is reduced in the present work. The high-level *ab initio* (MCSCF/MRCI level) methods with big basis set aVQZ are used to calculate 27690 potential energy points in the MOLPRO quantum chemistry package using the Jacobi coordinate. Meanwhile, the B-spline fit method is used to reduce the global potential energy surface in this present work. The shallow well complexes are found in the present work when the angles *θ* = 30°, 60°, and 90°. Analysing the global potential energy surfaces can get the conclusion that reactants should overcome at least 0.894 eV energy to cross the transition state and reach products. This study will be helpful for the analysis in histopathology and for the study of biological and medical mechanisms.

## 1. Introduction

As the most active element of halogens, fluorine often plays an important role in the chemical reaction process. In the chemical industry, fluorine is widely used in the fields of semiconductors and new energy, which is particularly critical for future development. Therefore, the three-dimensional potential energy surface of F_3_ is very interesting. Duggan and Grice have described the approximate shape of the potential energy surface of F_2_+F [1]. Artau et al. [2] determined the dissociation energy of the F_2_ bond by the collision-induced dissociation energy method.

Although many studies have been made on fluorine [1–8], while exchange reactions between halogen atoms and halogen atoms have been studied by many scholars [9–16], the exact understanding of the three-dimensional potential energy surface for F_3_ is so limited. Currently, quantum chemistry calculations are able to provide sufficiently reliable data for this system, and potential energy surfaces (PESs) can help us to understand possible reaction mechanisms and estimate reaction rates. The potential energy surface of F_3_ was studied using the classical orbital method combined with direct dynamic electronic structure calculation. By now, a train of scholars have studied the exchange reaction of trifluoride ions [2–4], but there is no precise dynamic study for this system. In order to accurately describe this system, three-dimensional PESs for the title system are constructed in this paper.

In the current work, the accurate three-dimensional PESs for this system are deduced. The details of this paper are shown as follows: the computational method will be introduced in the second chapter and the detailed description of PESs will be introduced in the third section. In the last part, the conclusion and discussion will be demonstrated.

## 2. Computational Methods

Three adiabatic potential energy points for F_3_ are calculated with MCSCF/MRCI method with aVQZ [17] basis sets. The lowest potential energy surface is described in this study. All the calculations are performed in the MOLPRO 2012 package [18]. A total of 12 (9A′ + 3A^″^) active orbitals are considered in the present work. And 225 (138A′ + 87A^″^) external orbitals are used in this work. Thus, the MCSCF function included 360 determinants and 792 intermediate states for the triatomic molecule system. In the MRCI calculated progress, 3 orbitals (3A′ + 0A^″^) are put in the core orbital, and 24 electrons are set in the valence space. The total number of contracted configurations is 595861.

**Figure 1:**
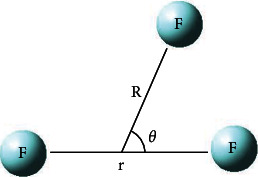
Jacobi coordinate for the F_3_(^2^A^″^) system.

The Jacobi coordinate (*r*, *R*, *θ*), which can express the three-dimensional PESs well in our previous works on triatomic systems [3, 19–23], is used in the present work. *r* is used to show the distance between two fluorine atoms, *R* demonstrates the distance from the reduced mass of the two fluorine atoms to the third fluorine atom, and the angle between these two vectors is presented by *θ* (see Figure 1). *R* is used in the range of 0.0 Å-20.0 Å with 69 points, *r* is fixed from 0.4 Å to 5.0 Å with 39 points using different step sizes, and the range of angles *θ* is 0°-90° with a 10° scan step. Therefore, in total, 27690 geometries are calculated. So the large number of scanning points that can warrant the quality of the following fitted the PESs. The three-dimensional B-spline method [24, 25] is used to get the procedure interpolate surface in the whole scan field.

## 3. Results and Discussion

The three-dimensional adiabatic potential energies of the lowest energy state for the F_3_(^2^A^″^) system is deduced in the present work; for a clearer understanding of the character of this system, the two-dimensional (2D) PES is shown in the following part. For a clearer discussion of these PESs, the energy of F+F_2_ is shifted to 0.00 eV.

The one-dimensional potential energy surface for F_2_ is studied in the present work. The dissociation energy is 1.40 eV, which compares well to the former theoretical and experimental results (1.25 eV and 1.60 eV, respectively) [2, 26].

The 2D lowest energy state adiabatic potential energy surfaces (2D-PESs) have been shown in different angles for the F_3_(^2^A^″^) energy surface. The four different angles (*θ* = 0°, 30°, 60°, and 90°) are used to describe the vivid change of the PES. The upper part of each figure is the 2D image PES, and the corresponding contour plots of each 2D-PES are shown in the lower part of each figure. In the contour plotted part, the difference of the energy between two adjacent curves is 0.20 eV; the short distance between two adjacent curves means big energy changes with distance (*R* or *r*) and vice verse. Some important parts (isomers or transition state parts)are presented in enlarged panel in each figure.

**Figure 2:**
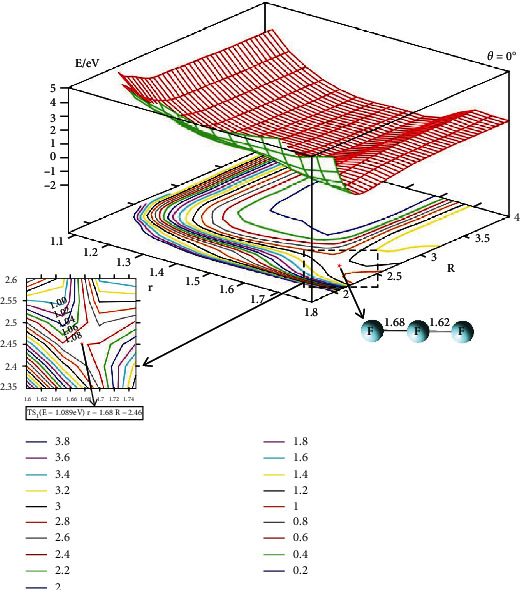
Adiabatic potential energy surface (in eV) and contour plots of the potential energy surface for the lowest energy state of F_3_(^2^A^″^) as the function of *r* and *R* (in Å) in C_∞_ symmetry.

The 2D-PES for F_3_(^2^A^″^) in linear symmetry is plotted in Figure 2. From this figure, it can be found that the potential energies increase with the decrease of the distance of the *R*. There is no minimum in this figure. The PES of the F_3_(^2^A^″^) system in linear symmetry is flat when the *r* is in the range of 1.3 Å < *r* < 1.6 Å and $R>3.0\;\overset{{}^{\circ}}{A}$. In this area, the energy is lower than 0.2 eV and larger than 0.0 eV, so this area is embraced by the blue curve (the blue curve is the contour line of the energy of 0.2 eV). An enlarged figure is plotted to show the transition state (TS) area. The geometry of this TS is $r=1.68\;\overset{{}^{\circ}}{A}$ and $R=2.46\;\overset{{}^{\circ}}{A}$. The linear symmetry of reactants F*_a_*+F*_b_*F*_c_* overcomes 1.089 eV energy crossing the TS then transforming to the products F*_a_*F*_b_*+F*_c_*.

**Figure 3:**
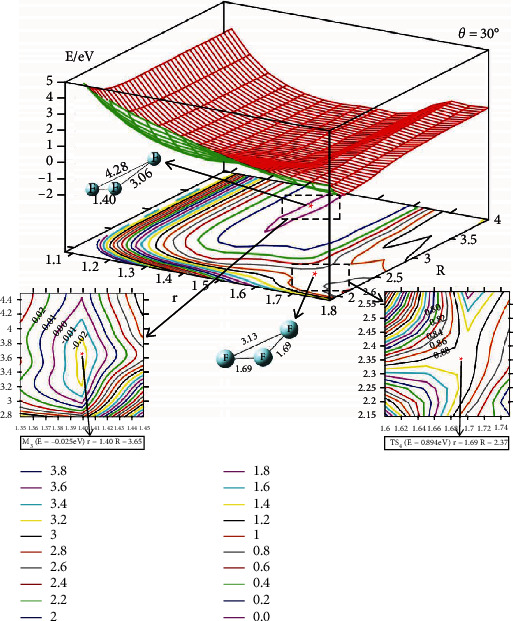
Adiabatic potential energy surface (in eV) and contour plots of the potential energy surface for the lowest energy state of F_3_(^2^A^″^) as the function of *r* and *R* (in Å) for *θ* = 30°. The important isomer and transition sate are shown in the lower panels.

The character of the PES for the *θ* = 30° is shown in Figure 3. There is a minimum in this angle, the geometry of the minimum is $r=1.40\;\overset{{}^{\circ}}{A}$, and $R=3.65\;\overset{{}^{\circ}}{A}$. The energy of this minimum is -0.025 eV, which is slightly lower than that of reactants. The difference of these two energies is so small that the minimum is not stable. The reaction pathway along the *θ* = 30°, and the reaction barrier is 0.88 eV.

**Figure 4:**
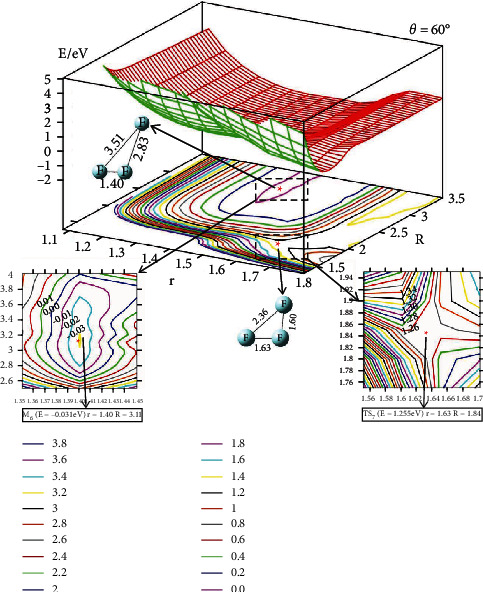
Adiabatic potential energy surface (in eV) and contour plots of the potential energy surface for the lowest energy state of F_3_(^2^A^″^) as the function of *r* and *R* (in Å) for *θ* = 60°. The important isomer and transition sate are shown in the lower panels.

The character of the PES with *θ* = 60° (see Figure 4) is similar to that of *θ* = 30°: Firstly, both of these two figures have a shallow minimum; the energy of the minimum for *θ* = 60° is -0.03 eV and the geometry of this minimum is $r=1.40\;\overset{{}^{\circ}}{A}$ and $R=3.11\;\overset{{}^{\circ}}{A}$. Secondly, the reactants want to change to the products through these two-angles reaction pathway and the additional energies should be given to this system to overcome the transition state. The energy of the TS for *θ* = 60° is 1.255 eV, which is higher than that of *θ* = 30°. The geometry of the TS is $r=1.63\;\overset{{}^{\circ}}{A}$ and $R=1.84\;\overset{{}^{\circ}}{A}$.

**Figure 5:**
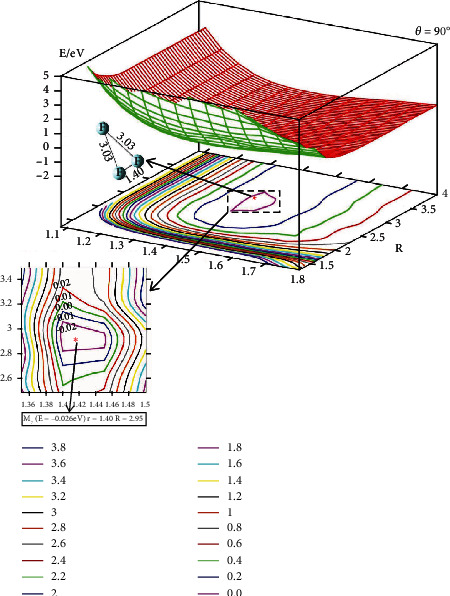
Adiabatic potential energy surface (in eV) and contour plots of the potential energy surface for the lowest energy state of F_3_(^2^A^″^) as the function of *r* and *R* (in Å) for C_2*v*_ symmetry. The importance isomer is shown in the lower panel.

Figure 5 shows the adiabatic PES of the title system for *θ* = 90°. When the F atom attacks the F_2_ molecule along *θ* = 90°, and for the same *r*, the energy is nearly unchanged with the function of *R* when $R>3.5\;\overset{{}^{\circ}}{A}$; when *R* is decreasing to 2.95 Å and $r=1.40\;\overset{{}^{\circ}}{A}$, the system reaches the shallow well complex; the energy of this complex is -0.026 eV. After this complex, the energy of this system becomes higher and higher, decreasing the *R* distance further.

The figures for *θ* = 10, 20, 40, 50, 70, and 80° also have been plotted in Figure S1-S6 (see the Supporting Information).

**Figure 6:**
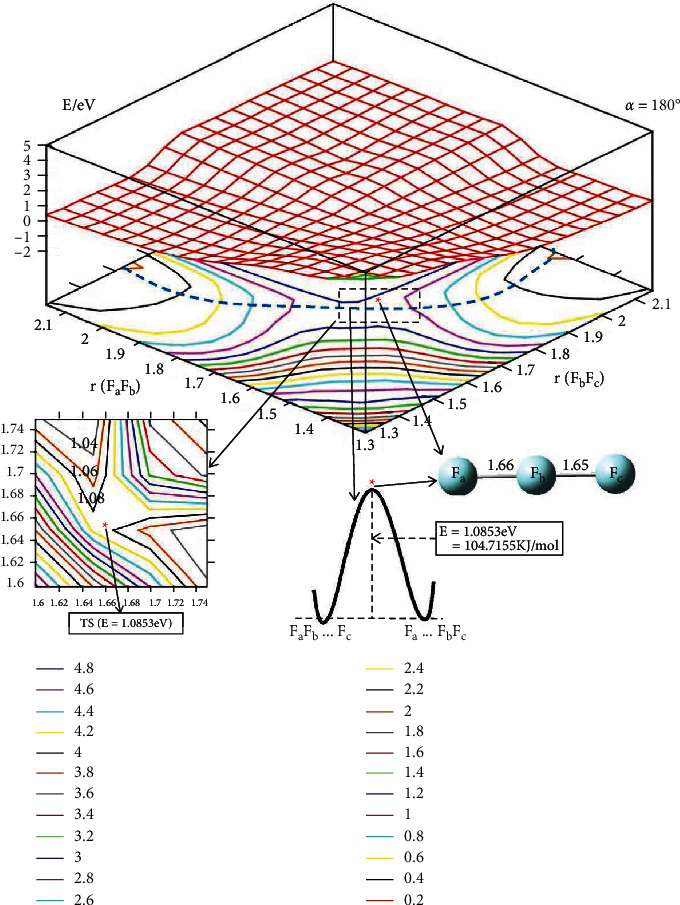
Adiabatic potential energy surface (in eV) and contour plots of the potential energy surface for the lowest energy state of F_3_(^2^A^″^) as the function of *r*(F_*a*_F_*b*_) and *r*(F_*b*_F_*c*_) (in Å) in C_∞_ symmetry.

The 2D-PES for F_3_(^2^A^″^)in the function with *r*(F_*a*_F_*b*_), *r*(F_*b*_F_*c*_) and *α* = 180° is plotted in Figure 6. The reaction barrier is 104.715 kJ/mol, this is good agreement with Duggan and Grice's work( 108 Kj/mol). The important region is a magnified plot in the same figure. The blue dotted line shows the easiest reaction pathway for the title reaction. Meanwhile, the sketch map of this dotted line is plotted in the lower panel, which shows that the reaction barrier is 104.715 kJ/mol; compared with Duggan and Grice's work (108 kJ/mol) [1], the results are almost consistent. The geometry of the transition state for the reaction pathway is *r*(F_*a*_F_*b*_) = 1.66 Å and *r*(F_*b*_F_*c*_) = 1.65 Å.

## 4. Conclusions

The electronic adiabatic state PESs for the lowest energy state of F_3_ are presented with the high-level *ab initio* (MCSCF/MRCI level) method by the MOLPRO quantum chemistry package in the Jacobi coordinate. The potential energies are calculated using the aVQZ basis set over a larger region of configuration space. 27690 potential energy points are calculated for getting the globally accurate PESs, which is deduced by an accurate B-spline fit method. There is no minimum in the PES with *θ* = 0.0°, but there is a transition state in this PES. The process of F*_a_*+F*_b_*F*_c_*→F*_a_*F*_b_*+F*_c_* along the angle *θ* = 0.0° should be given additional energy of 1.089 eV, but for *θ* = 30.0°, this energy is reduced to 0.894 eV; when the angle increases to 60°, the reaction barrier will reach 1.255 eV. There are shallow complexes in the PES for *θ* = 30°, 60°, and 90° and the energies are -0.025 eV, -0.031 eV, and -0.026 eV, respectively. The proposed method in this study will be helpful for the development of biological and medical mechanisms.

It is worth performing the full dynamical study with this global potential energy surface. We will continue this work in the following study.

## Data Availability

Data are available, which are included in the code.

## Supplementary Materials

Supplementary Materials The supporting information is available free of charge at BioMed Research International. The figures for *θ* = 10, 20, 40, 50, 70, and 80° also have been plotted in Figure S1-S6 (PDF).
