# Supplementary material for: The Reaction Mechanism Study for the F3 System
Source: Biomed Res Int. 2022 Apr 28;2022:7088063. doi: 10.1155/2022/7088063 (PMC9071887; doi:10.1155/2022/7088063)
Supplement: Supplementary Materials — The supporting information is available free of charge at BioMed Research International. The figures for θ = 10, 20, 40, 50, 70, and 80° also have been plotted in Figure S1-S6 (PDF). [file 7088063.f1.pdf]

Supporting Information

The Reaction Mechanisms study For the F_3 system

Dequan Wang[a*||], Nan Gao[c||], Hongmei Yu[d], Yuxuan Bai[e], Jing Cao[a], Chunmei Hu[b*], Yanchun Li[a], Huiling Liu[a], Xuri Huang[a]

[a]Laboratory of Theoretical and Computational Chemistry, Institute of Theoretical Chemistry, Collage of Chemistry, Jilin University, Changchun, People's Republic of China

[b]Hematology and Oncology Department, The Second Hospital, Jilin University, Changchun, People's Republic of China

[c]Department of Thoracic Surgery, China-Japan Union Hospital of Jilin University, Changchun, People's Republic of China

[d]High Performance Computing Center of Jilin University, People's Republic of China

[e]College of Life Science, Sichuan Agricultural University, Ya 'an, People's Republic of China

E-mails: dequan_wang@jlu.edu.cn; hucm@jlu.edu.cn

[||]these authors contributed equally to this work.

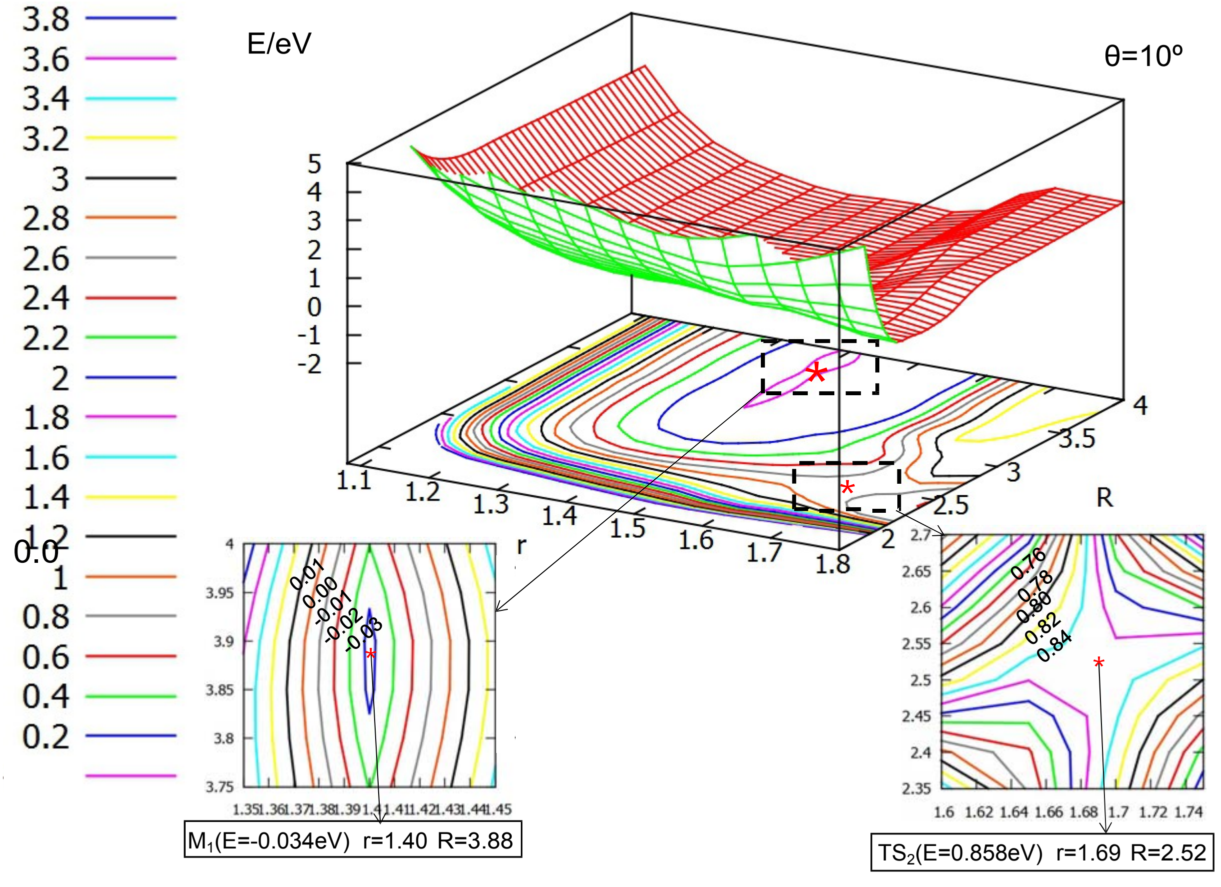

Figure S1: Adiabatic potential energy surface (in eV) and contour plots of the potential energy surface for the ground state of F_3 as the function of r and R (in Å) for $\theta = 10^{\circ}$. The importance isomer and transition sate are shown in the lower pannels.

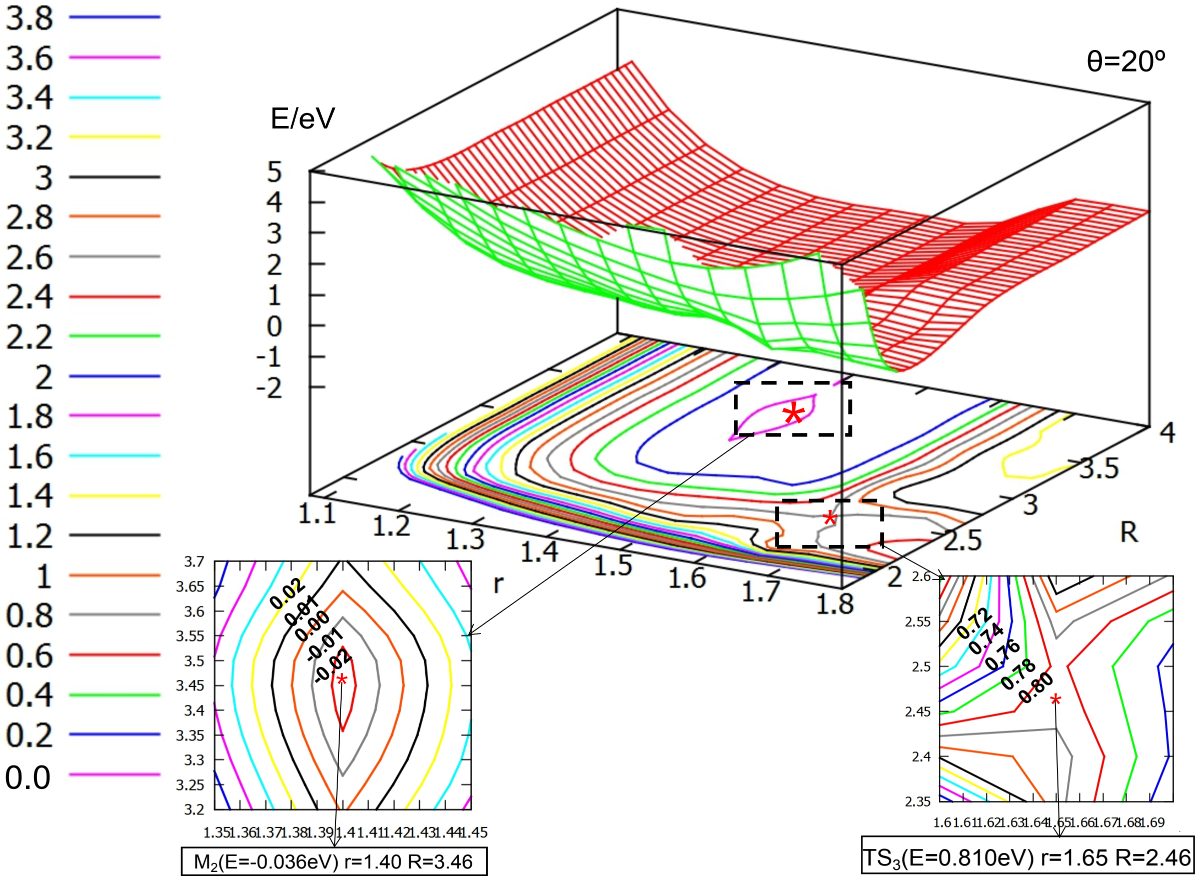

Figure 2: Adiabatic potential energy surface (in eV) and contour plots of the potential energy surface for the ground state of F_3 as the function of r and R (in Å) for $\theta = 20°$. The importance isomer and transition sate are shown in the lower pannels.

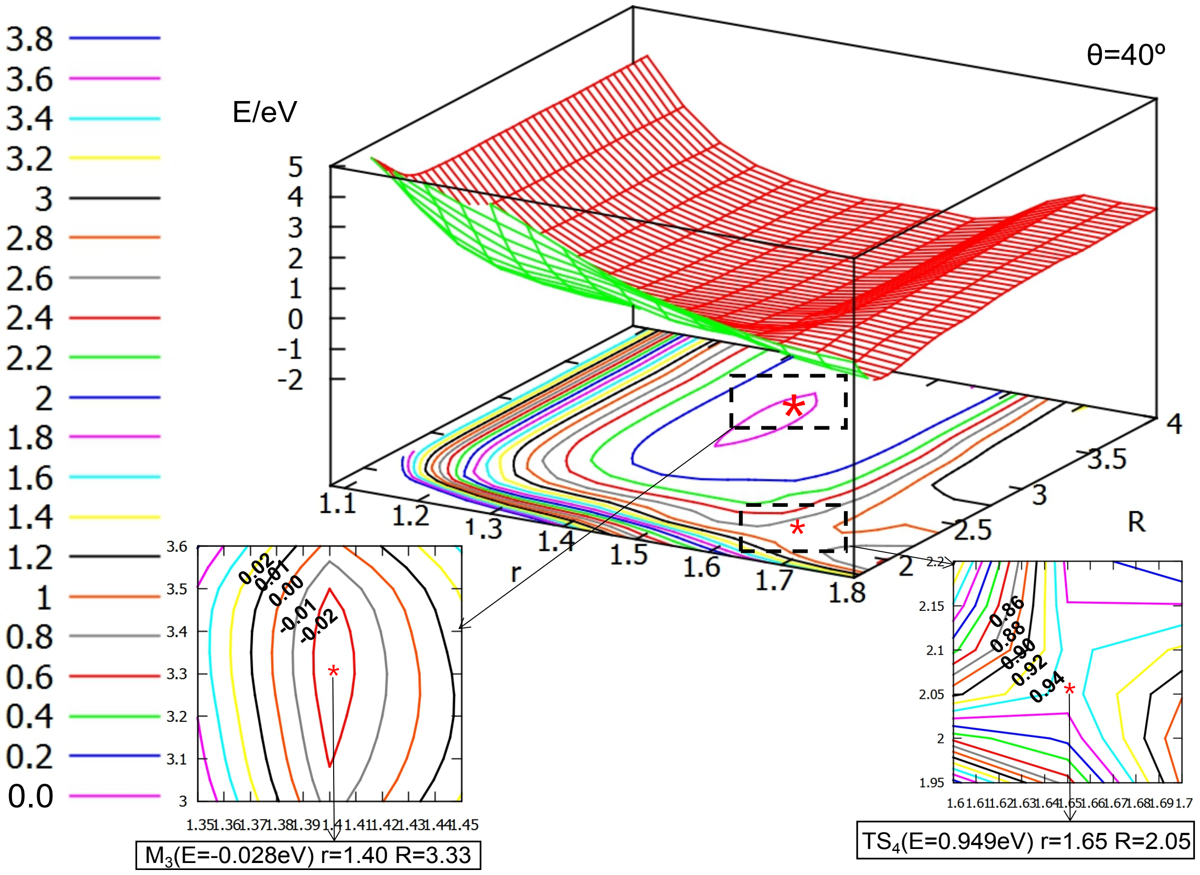

Figure 3: Adiabatic potential energy surface (in eV) and contour plots of the potential energy surface for the ground state of F_3 as the function of r and R (in Å) for $\theta = 40°$. The importance isomer and transition sate are shown in the lower pannels.

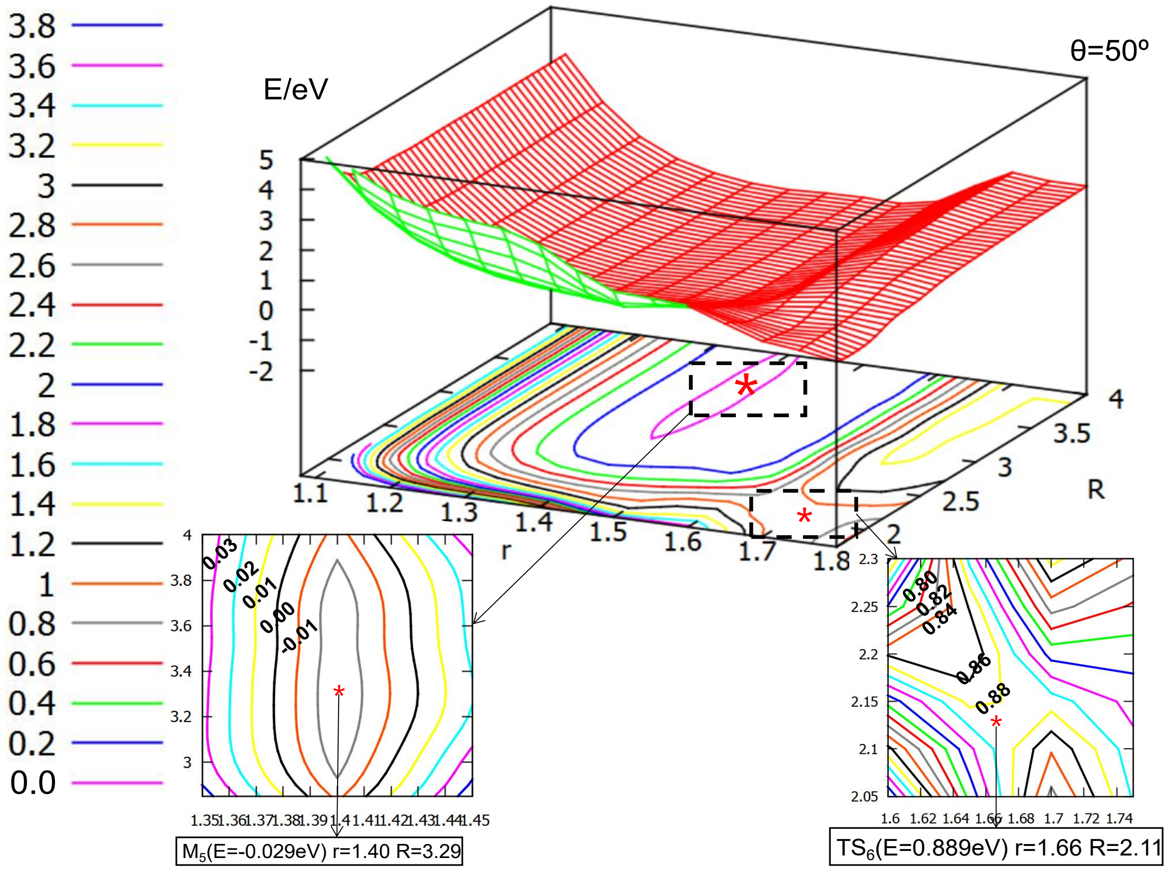

Figure 4: Adiabatic potential energy surface (in eV) and contour plots of the potential energy surface for the ground state of F_3 as the function of r and R (in Å) for $\theta = 50°$. The importance isomer and transition sate are shown in the lower pannels.

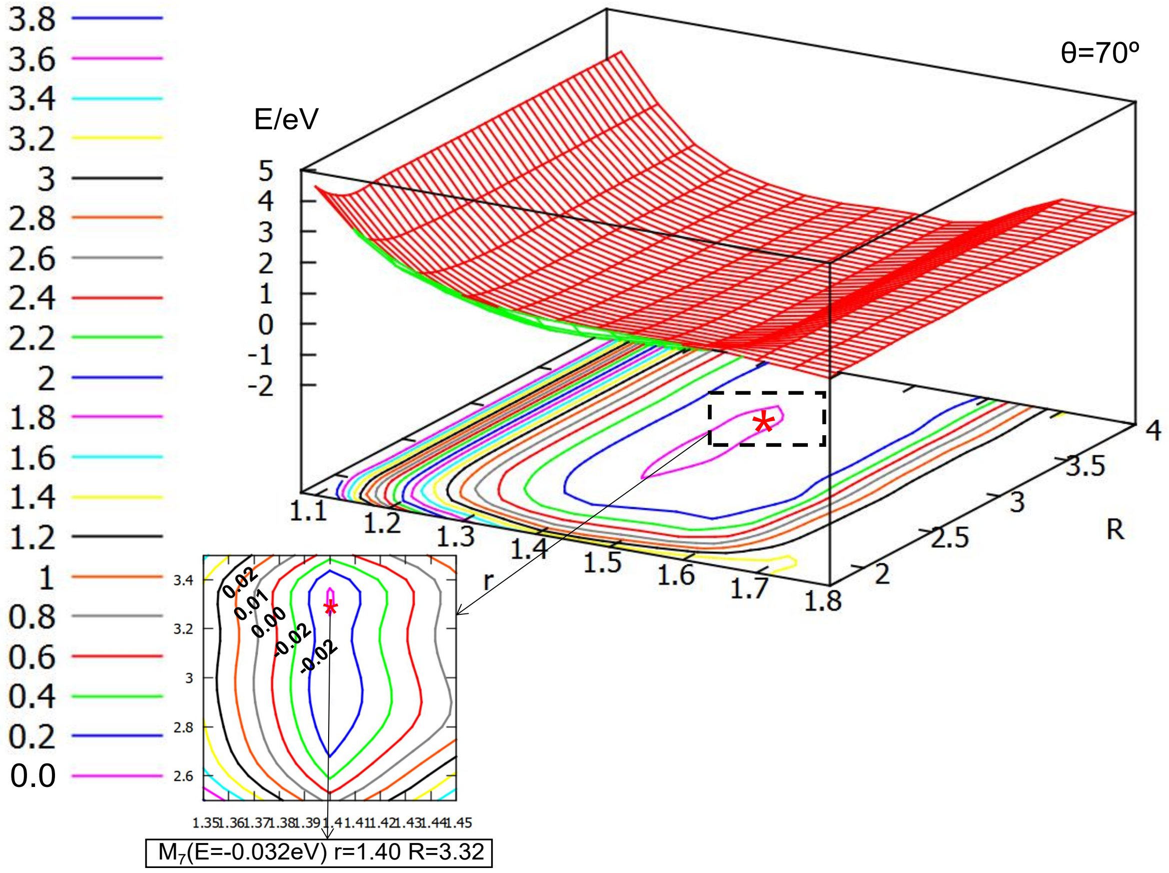

Figure 5: Adiabatic potential energy surface (in eV) and contour plots of the potential energy surface for the ground state of F_3 as the function of r and R (in Å) for $\theta = 70°$. The importance isomer is shown in the lower pannels.

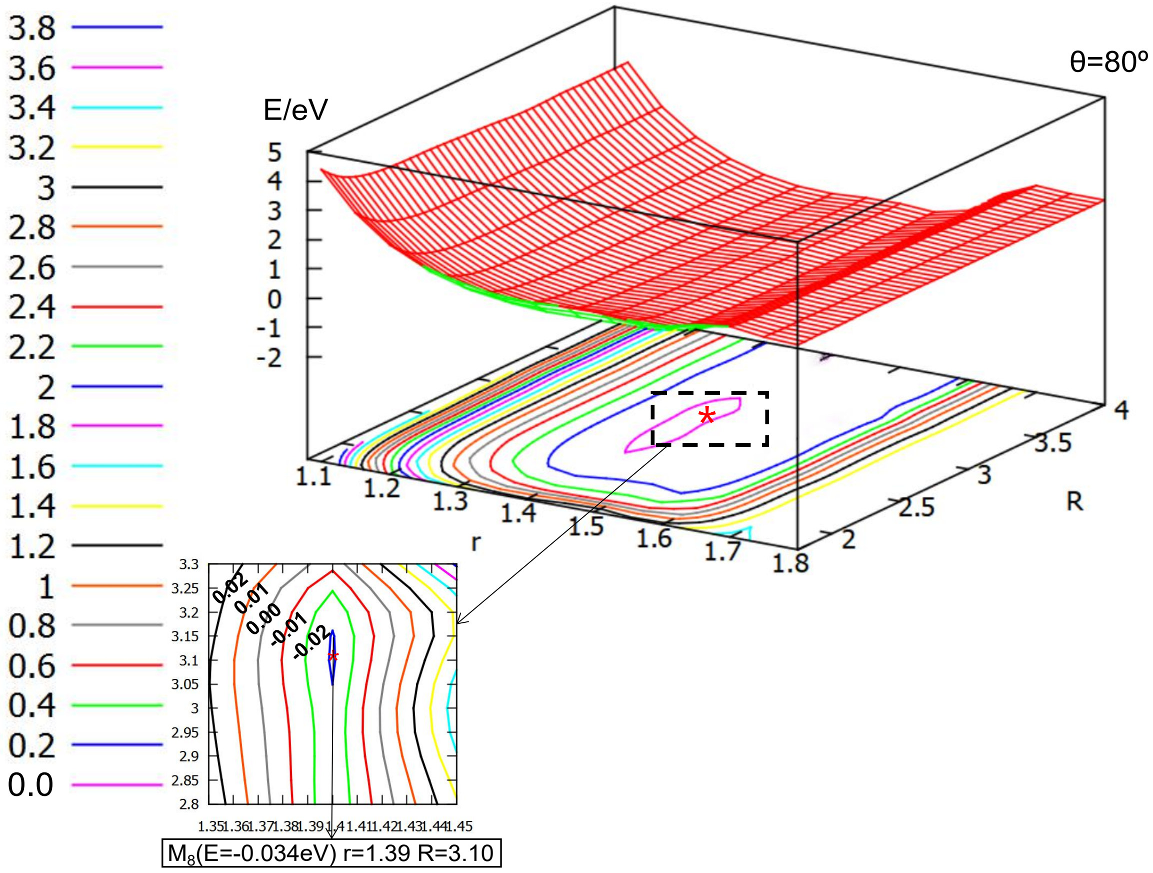

Figure 6: Adiabatic potential energy surface (in eV) and contour plots of the potential energy surface for the ground state of F_3 as the function of r and R (in Å) for $\theta = 80°$. The importance isomer is shown in the lower pannels.
